# Supplementary figures and images for: The Lid/KDM5 histone demethylase complex activates a critical effector of the oocyte-to-zygote transition
Source: PLoS Genet. 2020 Mar 5;16(3):e1008543. doi: 10.1371/journal.pgen.1008543 (PMC7058283; doi:10.1371/journal.pgen.1008543)

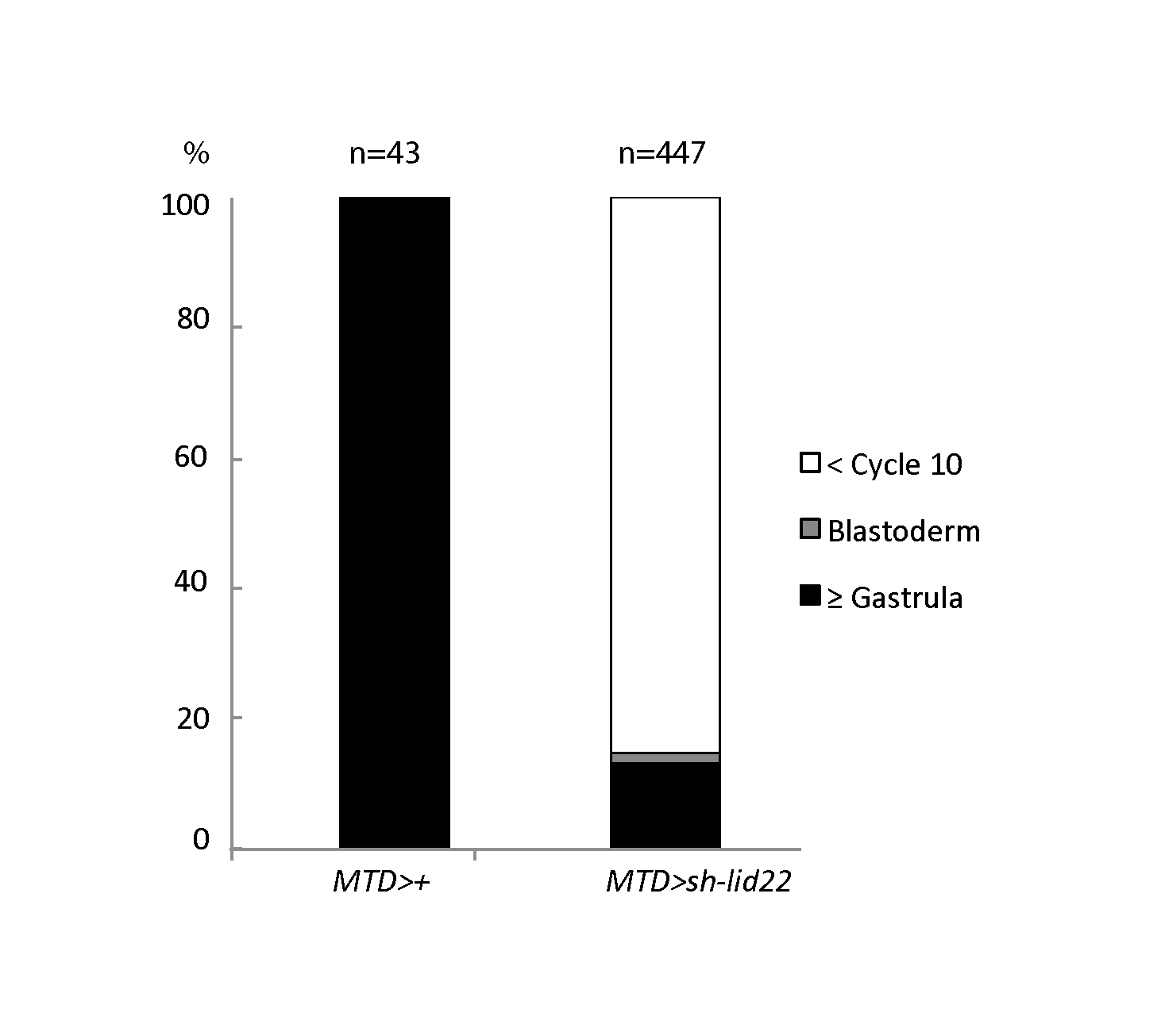

Supplement: S1 Fig — Embryos were collected for four hours and aged for another four hours at 25°C before DAPI staining and examination in fluorescent microscopy. More than 85% of lid KD embryos arrest development before the blastoderm stage. In contrast, 100% of control embryos had reached gastrula or later stages. (TIF) [file pgen.1008543.s001.tif]

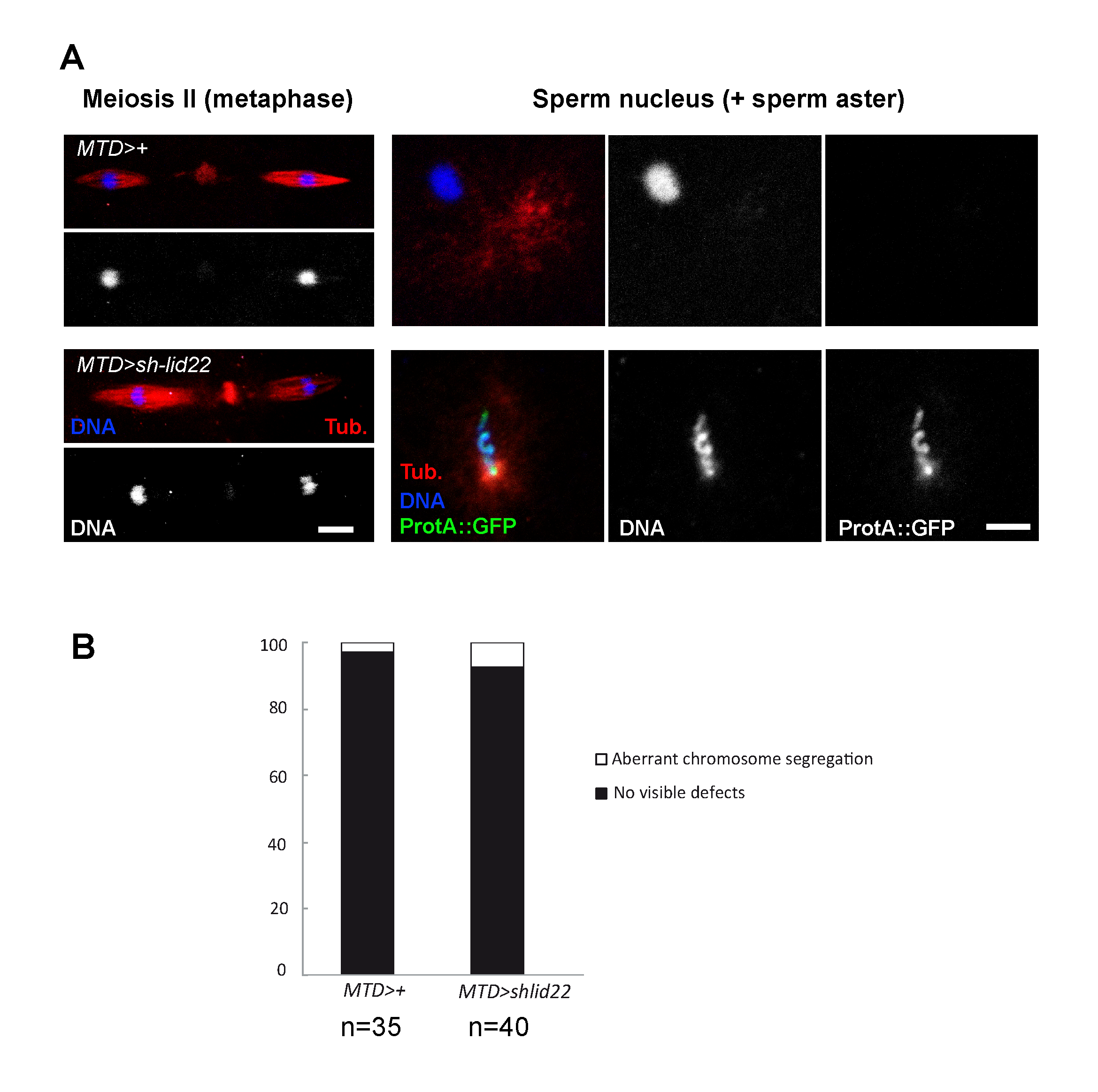

Supplement: S2 Fig — A—Representative confocal images of eggs in metaphase of meiosis II stained for DNA (blue), alpha-tubulin (red) and ProtA::GFP (green). The tandem of meiotic spindles is shown on the left, the corresponding male nucleus from the same egg is on the right. Bars: 5 μm. B—Quantification of meiosis II phenotypes (normal or abnormal chromosome segregation). (TIF) [file pgen.1008543.s002.tif]

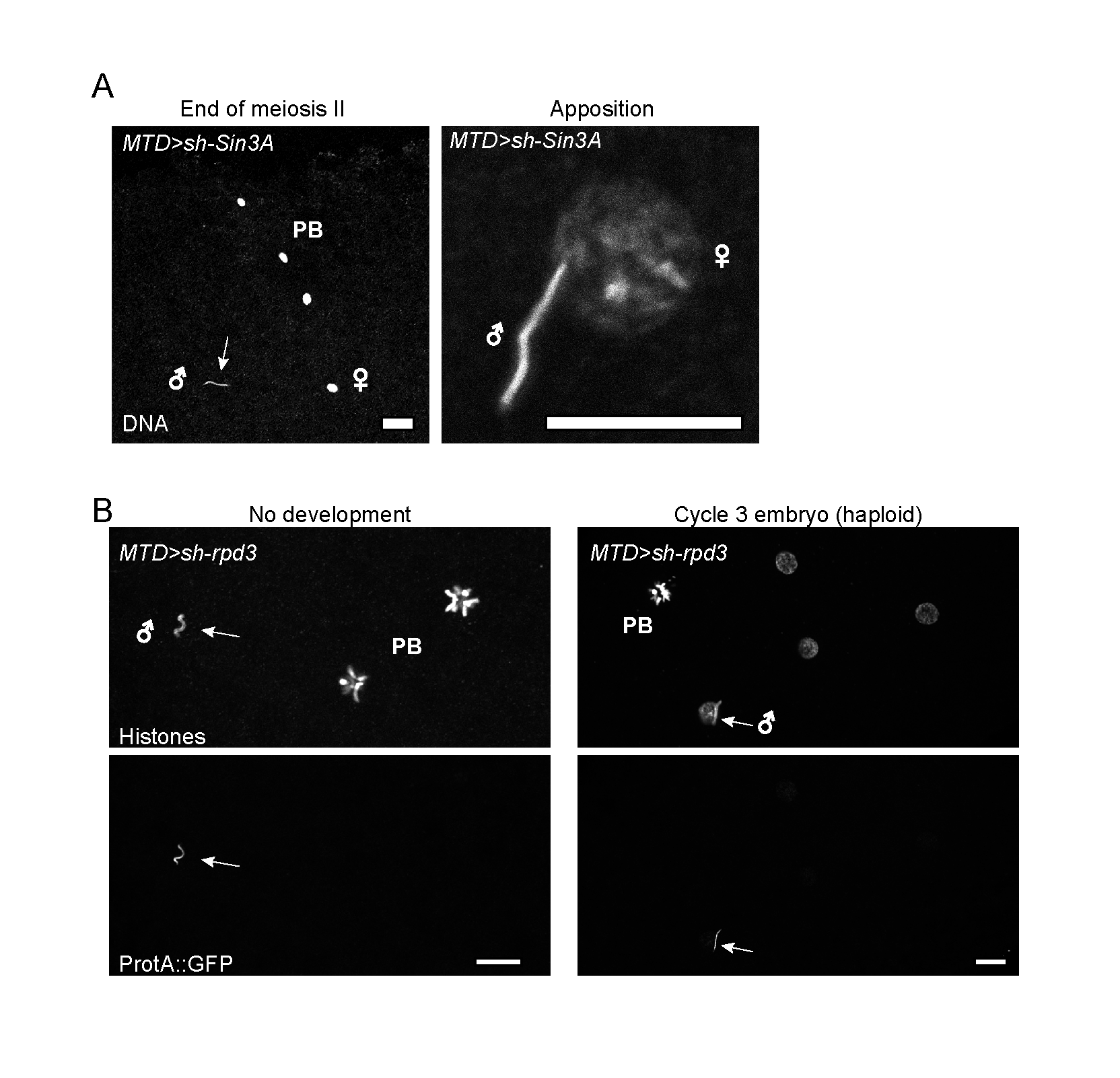

Supplement: S3 Fig — A—Confocal images of Sin3A KD eggs stained for DNA at the indicated stages. The sperm nucleus in the left panel is indicated (arrow). Bar: 10 μm. PB: Polar bodies. B—Confocal images of rpd3 KD early embryos (from ProtA::GFP fathers) stained for DNA and anti-GFP. The sperm nucleus is indicated (arrows). Bar: 10 μm. PB: Polar bodies. (TIF) [file pgen.1008543.s003.tif]

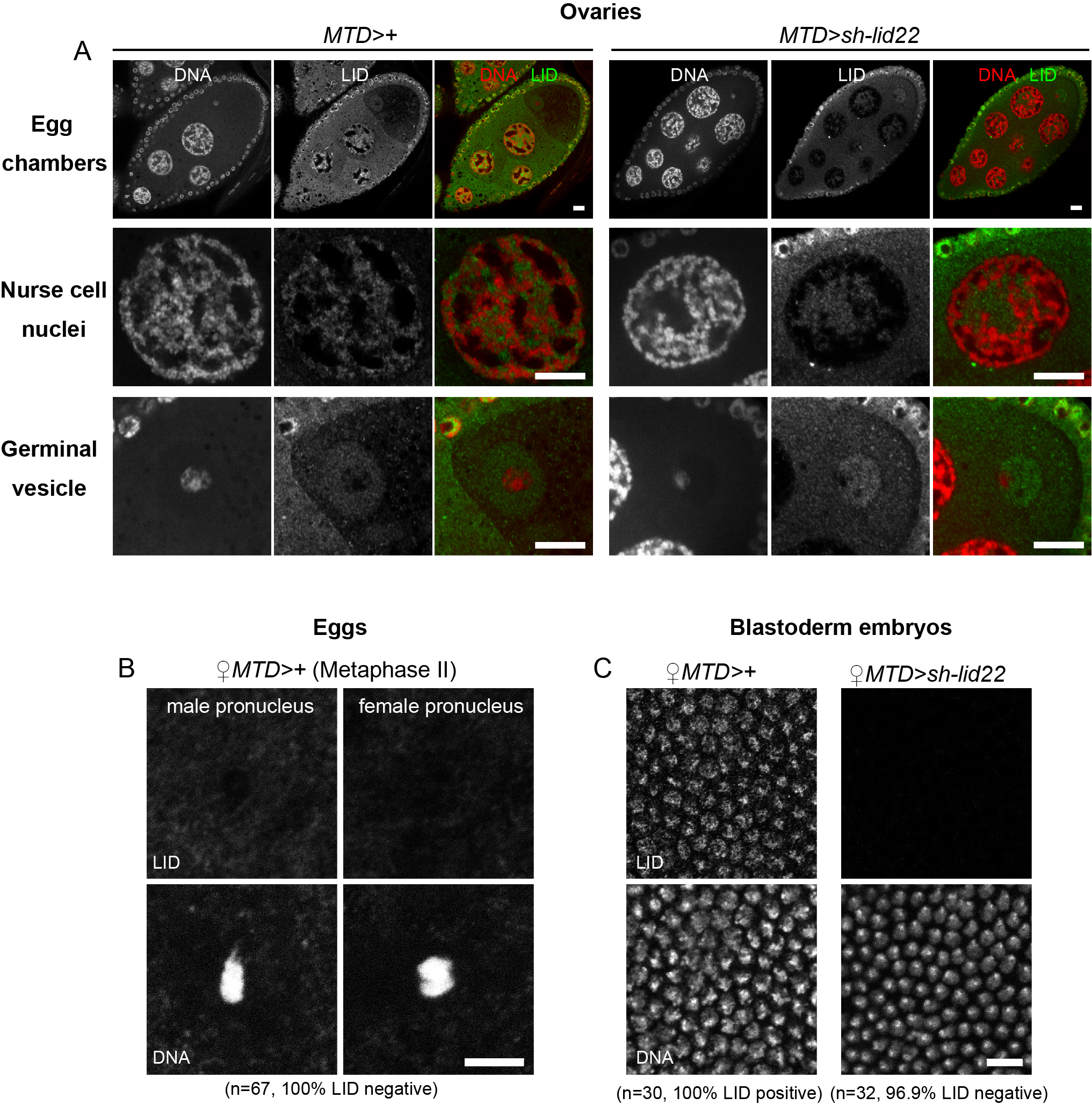

Supplement: S4 Fig — A—Top row: confocal images of stage 10 egg chambers from control (left) and lid KD (right) females stained for DNA (red) and anti-Lid (green). Middle row: detail of a nurse cell nucleus. Bottom row: detail of the oocyte germinal vesicle (oocyte nucleus). Bar: 20 μm. B—Confocal images of the male pronucleus and the female pronucleus from a control egg in meiosis II stained for DNA and anti-Lid. Bar: 10 μm. Quantification of Lid positive nuclei is indicated. C—Confocal images of a control (left) and lid KD (right) blastoderm embryo with same staining as in B. Bar: 10 μm. Quantifications of embryos with a positive/negative nuclear Lid staining are indicated for each genotype. (JPG) [file pgen.1008543.s004.jpg]

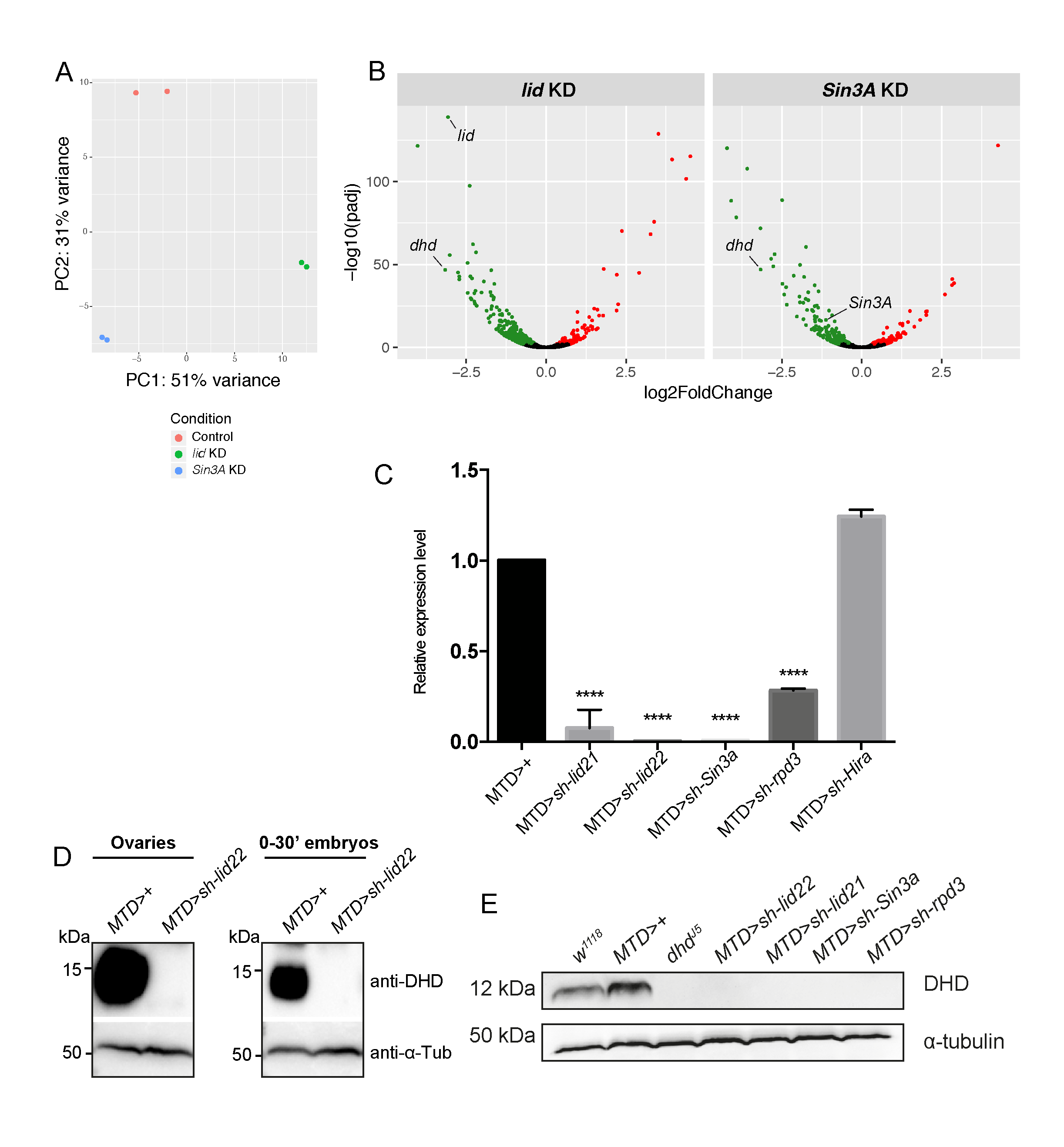

Supplement: S5 Fig — A—Principal Component Analysis of Control, lid KD and Sin3A KD ovarian transcriptomes (two biological replicates for each genotype). B—Volcano plot representations of Differentially-Expressed genes in Control vs lid KD (left) and Control vs Sin3A KD (right). C—RT-qPCR quantification of dhd mRNA levels in ovaries of indicated genotypes. mRNA levels were normalized to rp49 and shown as relative expression in MTD>+ control. Error bars represent SD (Dunnett’s multiple comparisons test to the control MTD>+, **** P < 0.0001). D—Western blot analysis of DHD in adult ovaries (left) and 0-30min postfertilization embryos (right). α-tubulin was used as a loading control. E—Western blot analysis of DHD in adult ovaries of indicated genotypes. α-tubulin was used as a loading control. (TIF) [file pgen.1008543.s005.tif]

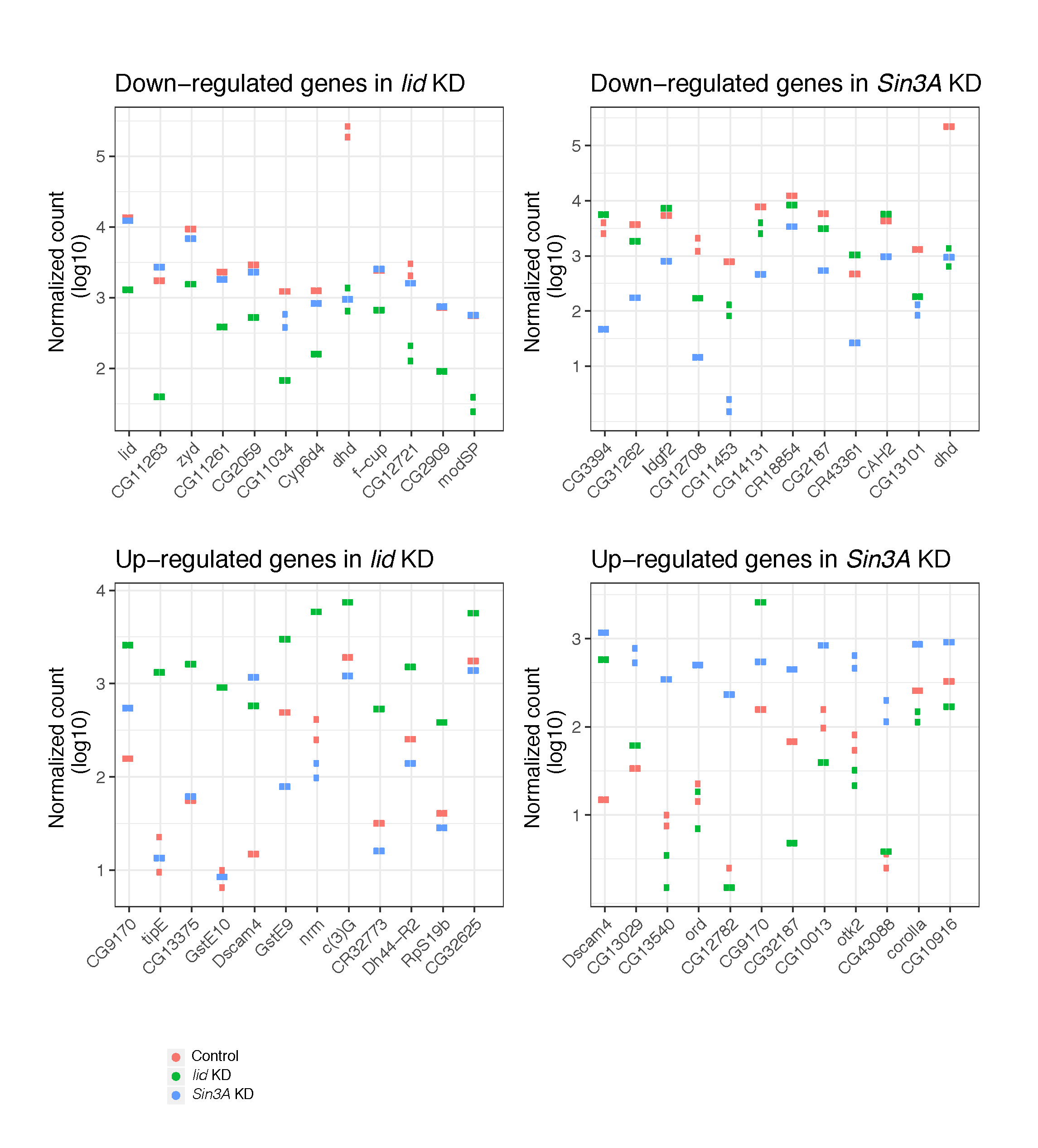

Supplement: S6 Fig — (TIF) [file pgen.1008543.s006.tif]

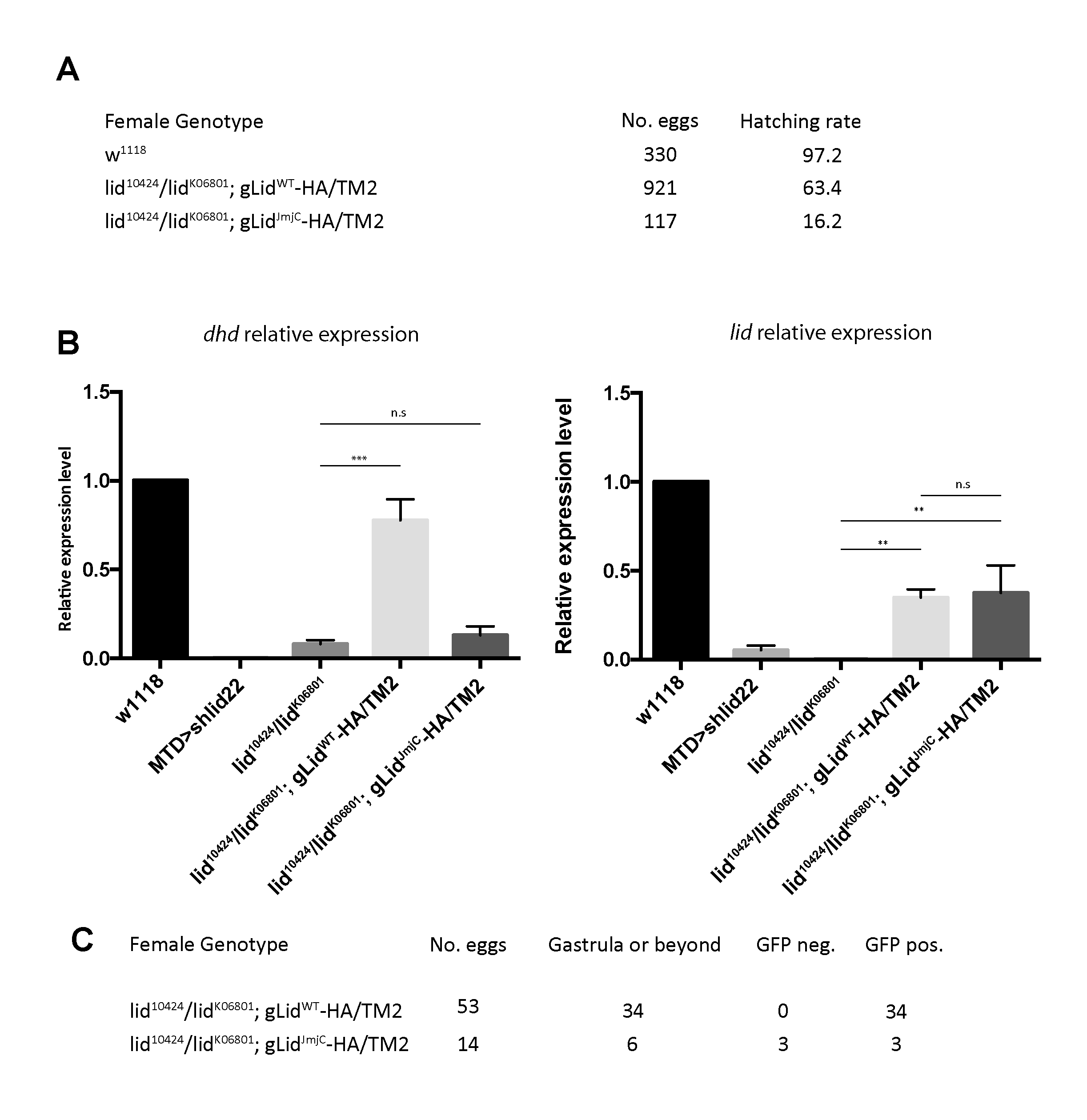

Supplement: S7 Fig — A—Embryo hatching rates from females of indicated genotypes. B—RT-qPCR quantification of dhd (left) and lid (right) mRNA levels in ovaries of indicated genotypes. mRNA levels were normalized to rp49 and shown as relative expression in w1118 control. Error bars represent SD (Dunnett’s multiple comparisons test to the control (** P <0.01; *** P = 0.0002). C—Analysis of paternal GFP::Cid expression in late embryos from indicated females (as in Fig 1B). (TIF) [file pgen.1008543.s007.tif]

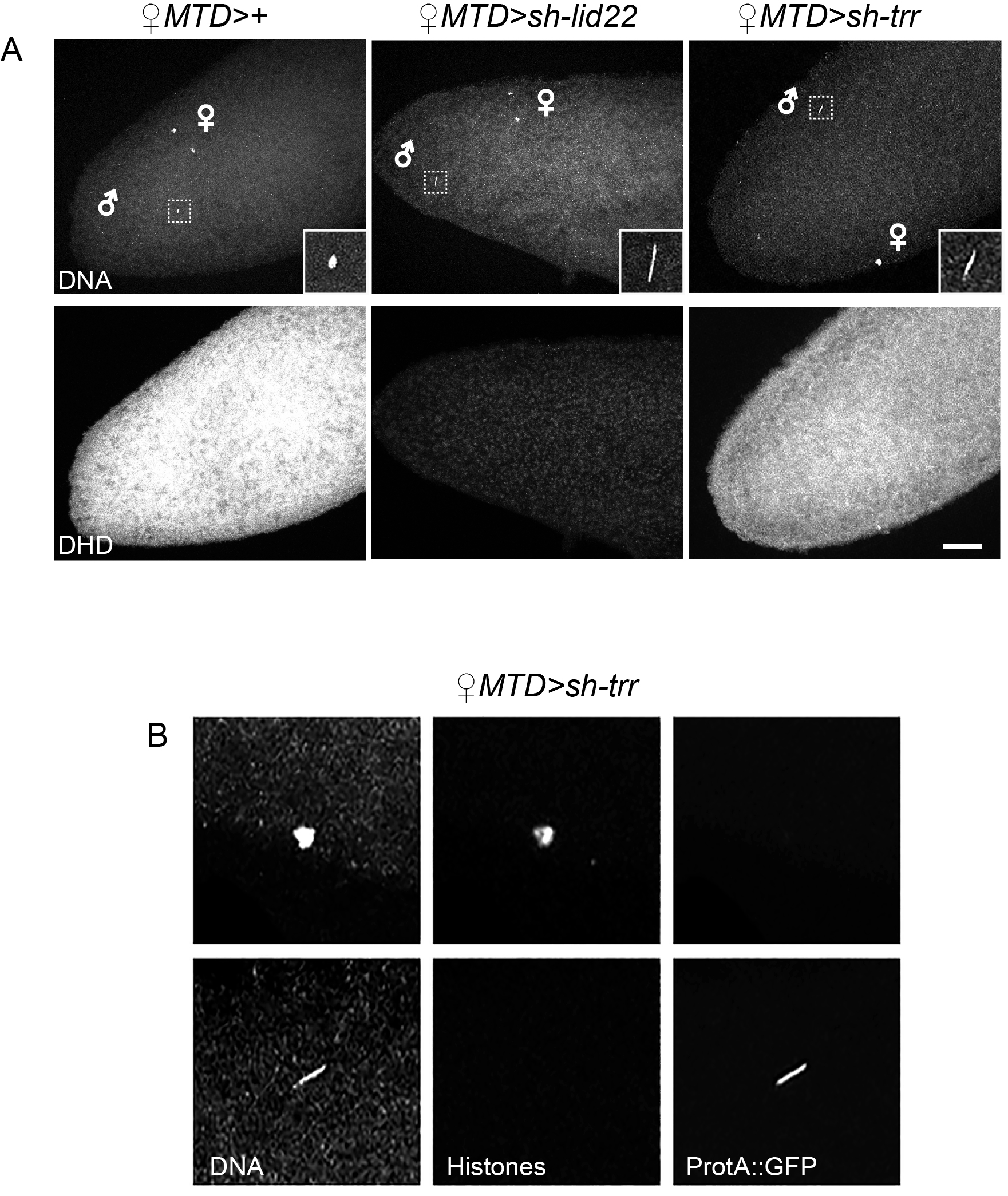

Supplement: S8 Fig — A—Confocal images of representative embryos of the indicated genotypes stained for DNA and anti-DHD. The fertilizing sperm nucleus is magnified in insets. Bar: 20 μm. B—Details of maternal chromosomes (top row) and sperm nucleus (bottow row) from a representative trr KD egg stained for ProtA::GFP and histones. (JPG) [file pgen.1008543.s008.jpg]
